# Comparison of DNA–Gold Nanoparticle Conjugation Methods: Application in Lateral Flow Nucleic Acid Biosensors

**DOI:** 10.3390/molecules28114480

**Published:** 2023-06-01

**Authors:** Qiaoling Ding, Wanwei Qiu, Chunxue Sun, Hongxin Ren, Guodong Liu

**Affiliations:** 1 School of Food Engineering, Anhui Science and Technology University, Fengyang 233100, China; 2 Yangtze Delta Drug Advanced Research Institute, No.100, Dongtinghu Road, Nantong 226133, China; 3 Enfin Biotech (Jiangsu) Co., Ltd., No.100, Dongtinghu Road, Nantong 226133, China; 4 School of Life and Health Science, Anhui Science and Technology University, Fengyang 233100, China; 5 School of Chemistry and Chemical Engineering, Linyi University, Shuangling Road, Linyi 276000, China

**Keywords:** DNA, gold nanoparticle, butanol dehydration, lateral flow, nucleic acid biosensor

## Abstract

Lateral flow nucleic acid biosensors (LFNABs) have attracted extensive attention due to their rapid turnaround time, low cost, and results that are visible to the naked eye. One of the key steps to develop LFNABs is to prepare DNA–gold nanoparticle (DNA–AuNP) conjugates, which affect the sensitivity of LFNABs significantly. To date, various conjugation methods—including the salt-aging method, microwave-assisted dry heating method, freeze–thaw method, low-pH method, and butanol dehydration method—have been reported to prepare DNA–AuNP conjugates. In this study, we conducted a comparative analysis of the analytical performances of LFNABs prepared with the above five conjugation methods, and we found that the butanol dehydration method gave the lowest detection limit. After systematic optimization, the LFNAB prepared with the butanol dehydration method had a detection limit of 5 pM for single-strand DNA, which is 100 times lower than that of the salt-aging method. The as-prepared LFNAB was applied to detect miRNA-21 in human serum, with satisfactory results. The butanol dehydration method thus offers a rapid conjugation approach to prepare DNA–AuNP conjugates for LFNABs, and it can also be extended to other types of DNA biosensors and biomedical applications.

## 1. Introduction

Lateral flow nucleic acid biosensors (LFNABs) have attracted extensive attention owing to their uncomplicated usage, low price, rapid on-site testing, short assay time, and results that are visible to the naked eye [1,2,3], and they are widely used in primary diagnosis, environmental pollutant detection, and food safety inspection [4,5,6]. One of the key steps to develop LFNABs is to prepare DNA–label conjugates, which affect the sensitivity of LFNABs significantly. Various labels (including gold nanoparticles (AuNPs) [2,7], fluorescent probes [8], dye-doped latex beads [9], carbon nanotubes [10], gold nanorods [11], gold–platinum nanoflowers [12], fluorescent carbon nanoparticles [13], quantum dots [14], etc.) have been used to construct LFNABs for nucleic acid detection. AuNPs are among the most frequently used labels, due to their special physicochemical properties and biocompatibility [15,16,17,18,19]. AuNP–DNA conjugates have a spherical nanostructure that attaches nucleic acid chains to the surface of AuNPs through covalent interactions between Au and S [20,21]. This usually consists of a nanoparticle core and a nucleic acid shell in close proximity, with covalently linked, densely functionalized, and strongly aligned nucleic acids [22,23]. AuNP–DNA conjugates can be used in chemistry, molecular diagnostics, gene regulation, and medicine in a number of different ways [24,25,26,27,28,29].

Various conjugation methods have been reported to immobilize DNA probes on the surface of AuNPs [30,31,32,33,34,35,36]. The most common method of binding DNA probes to AuNPs is the salt-aging method [30,31], which involves the slow addition of NaCl. This method takes two days to complete and is prone to agglomeration during the preparation process. Our group and others have modified this conventional salt-aging method with the help of dATP and reduced the whole process time to three hours, but still with great caution to avoid NP aggregation [32]. To overcome the aggregation issue, reduce the conjugation time, and increase the immobilization efficiency of DNA on the AuNP surface, a few new conjugation methods—including the low-pH method [33], freeze–thaw method [34], butanol dehydration method [35], and microwave-assisted dry heating method [36]—have been developed. Liu et al. reported a low-pH method to complete the AuNP–DNA conjugation process within a few minutes [33]. The low-pH conjugation environment reduces the electrostatic repulsion of the involved materials and avoids the aggregation issue. A freeze–thaw method was introduced by the same group and offered a highly efficient (20−30% more DNA) and rapid (minutes to hours) AuNP–DNA conjugation process [34]. Hao et al. reported a butanol dehydration method, which is contingent upon the swift removal of water from a DNA/AuNP blend in contact with a butanol phase [35]. The thiol-modified DNA was mixed with AuNPs, and the DNA probe was affixed to the surface of the AuNPs through immobilization by adding an appropriate amount of butanol to remove the water within a few seconds. Compared to other DNA conjugation strategies, the butanol dehydration method can increase the density of DNA by more than threefold. Recently, Huang et al. introduced a microwave (MW)-assisted heating dry method to immobilize DNA on the surface of AuNPs within a few minutes [36]. All recent DNA–AuNP conjugation methods reported in the literature have their advantages over the salt-aging method, but the applications using the AuNP–DNA conjugates have not been explored—particularly in the LFNAB research field.

In this study, three single-strand DNA probes (control DNA (Con-DNA), capture DNA (Cap-DNA), and detection DNA (Det-DNA)) were used to prepare LFNABs, and Det-DNA was immobilized on the AuNPs’ surface with the abovementioned five conjugation methods (salt-aging, low-pH, freeze–thaw, butanol dehydration, and microwave-assisted dry heating). Another single-strand DNA, which was complementary with both Det-DNA and Cap-DNA, was used as a model DNA target. We assessed and contrasted the analytical capabilities of the LFNABs and found that the LFNAB based on the butanol dehydration method offered the highest sensitivity. After experimental optimization, this LFNAB was capable of detecting a minimum amount of 5.0 pM single-strand target DNA without signal amplification, which is 100 times lower than that of the salt-aging method. The prepared LFNAB showed satisfactory results in the detection of miRNA-21 in human serum.

## 2. Results and Discussions

### 2.1. Performance Comparison of LFNABs Prepared with Five DNA–AuNP Conjugation Methods

**Scheme 1:**
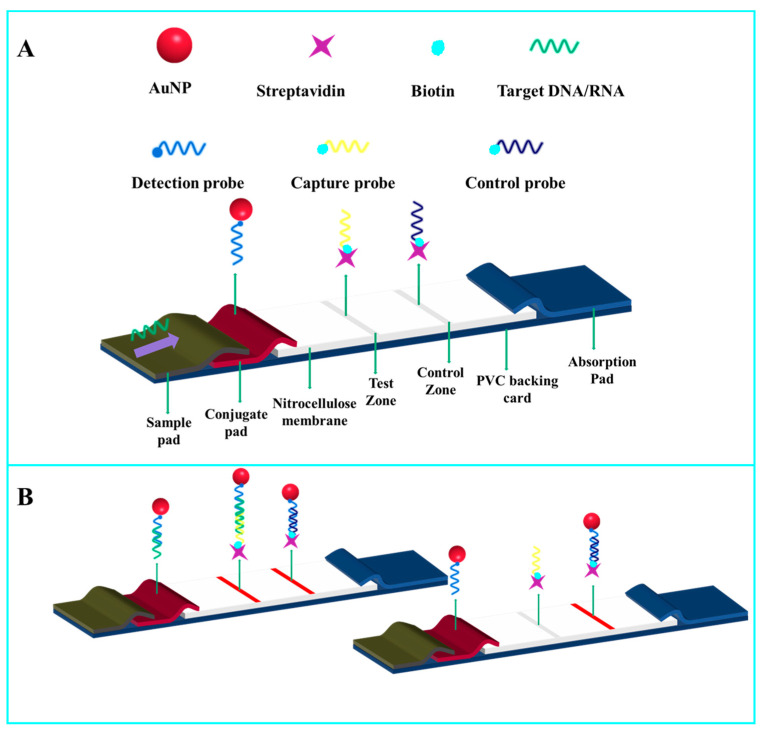
(**A**) Schematic illustration of the lateral flow nucleic acid biosensor; (**B**) the principle of visual detection in the presence (**left**) and absence (**right**) of the LFNAB.

The principle of DNA detection with LFNABs is based on an on-strip sandwich-type DNA hybridization, which captures AuNPs in the test zone (TZ) and control zone (CZ) for visual detection (Scheme 1). Three single-strand DNA probes were used to construct the LFNABs. In simpler terms, the Det-DNA and Cap-DNA probes match with the target DNA at both ends, while the Con-DNA completely matches with the Det-DNA. As shown in Scheme 1A, the thiol-modified Det-DNA probe was immobilized on the AuNPs, and the formed AuNPs–Det-DNA conjugate was sprayed on the conjugate pad. The TZ and CZ were prepared by dispensing SA–biotin–Cap-DNA and SA–biotin–Con-DNA complexes on the NC membrane, respectively. If the target DNA exists, the AuNPs–Det-DNA conjugates are captured by the two DNA–DNA hybridization reactions between AuNPs–Det-DNA and target DNA, and Cap-DNA hybridize with the other end of target DNA. The process of accumulating AuNPs in the TZ leads to the formation of a visual detection signal in the form of a red band (Scheme 1B). Meanwhile, the excess AuNP–DNA conjugates continue to migrate towards the control zone, where they are eventually captured by the Con-DNA. The captured AuNPs in the control zone show another red band, indicating that the LFNAB is functioning correctly. There is only one red band in the control zone when no target exists (Scheme 1B).

**Figure 1:**
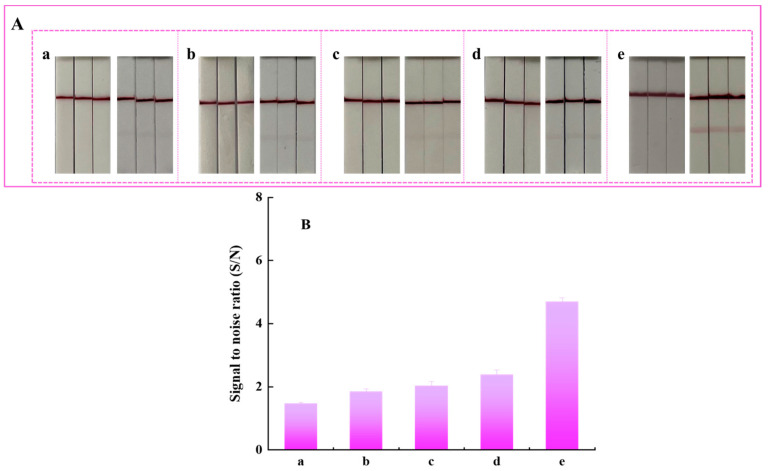
(**A**) Photo images of LFNABs after testing 0 pM (**left**) and 100 pM target DNA (**right**): (**a**) salt-aging method; (**b**) low-pH method; (**c**) freeze–thaw method; (**d**) microwave dry heating method; (**e**) butanol dehydration method. (**B**) The corresponding signal-to-noise ratios for the test bands of the LFNABs. Target DNA concentration: 100 pM; assay time: 15 min. Error bars indicate the standard deviations of three independent measurements.

We compared the performances of the LFNABs under five methods of preparing AuNP–DNA conjugates. Figure 1A presents images of the strips after testing 0 pM (left, control) and 100 pM target DNA (right). Each test was repeated three times, and a smartphone was used to take photos of the LFNABs after 15 min of assay time. The gray value of the test lines was determined by processing the images with ImageJ software (v1.8.0.172). All control tests showed no band in the T zones, indicating that no nonspecific adsorption occurred. Weak bands were observed in the T zones of the LFNABs prepared with the salt-aging method (a), low-pH method (b), freeze–thaw method (c), and microwave dry heating method (d). Bright red bands were shown in the test zones of LFNABs prepared with the butanol dehydration method. Figure 1B presents the corresponding signal-to-noise ratios for the test bands of the LFNABs. The highest S/N was obtained with the butanol dehydration method. Therefore, the butanol dehydration method was chosen to prepare AuNP–DNA conjugates for the LFNABs. AuNPs–Det-DNA conjugates prepared with the butanol dehydration method were characterized by UV–visible spectra and agarose gel electrophoresis (Figure S1). The red shift of the absorption peak of AuNPs in the UV–visible spectrum and the red band in the gel image indicated that Det-DNA was attached to the AuNPs’ surface successfully.

The detection limits, sensitivities and linear ranges of LFNABs prepared with the five methods are listed in the Supplementary Materials (Table S1). It can be seen that the butanol dehydration method showed the best analytical performance. Such difference may have been caused by the experimental conditions of the conjugation procedures. When AuNP–DNA conjugates were prepared by the salt-aging method, the addition of NaCl with increasing salt concentration led to a decrease in the ionic affinity of the probe, easily causing the phenomenon of agglomeration. In the process of conjugation, a large number of nonspecific adsorbed Det-DNA probes and non-conjugated AuNPs would inevitably exist, resulting in low coupling efficiency, which reduced the purity and specificity of the conjugates. When AuNP–DNA conjugates were prepared by the low-pH method, the AuNPs were prone to aggregation at low pH, resulting in increased particle size and unstable dispersion. Moreover, under acidic conditions, the protonation of carboxyl groups on the surface of AuNPs increases, leading to a decrease in coupling efficiency and a lower number of coupled DNA molecules, which affected the sensitivity and specificity of detection. When AuNP–DNA conjugates were prepared by the freeze–thaw method, the freeze–thaw process subjected the conjugate solution to mechanical cutting and ice crystal impacts, resulting in a decrease in the dispersion and stability of the coupling, which affected the detection sensitivity. When AuNP–DNA conjugates were prepared by the microwave-assisted dry heating method, the microwave heating process led to uneven heating and overheating, causing poor formation of conjugates and affecting the accuracy of the test results. When AuNP–DNA conjugates were prepared by the butanol dehydration method, hydroxyl groups were generated due to butanol dehydration, and these hydroxyl groups could interact with the carboxyl groups on the surface of the AuNPs to enhance the binding stability between the AuNPs and nucleic acids. By dehydrating butanol, the binding between AuNPs and nucleic acids can be achieved quickly and effectively, and this method is simple, fast, and easy to learn and use.

### 2.2. Optimization of Experimental Parameters

**Figure 2:**
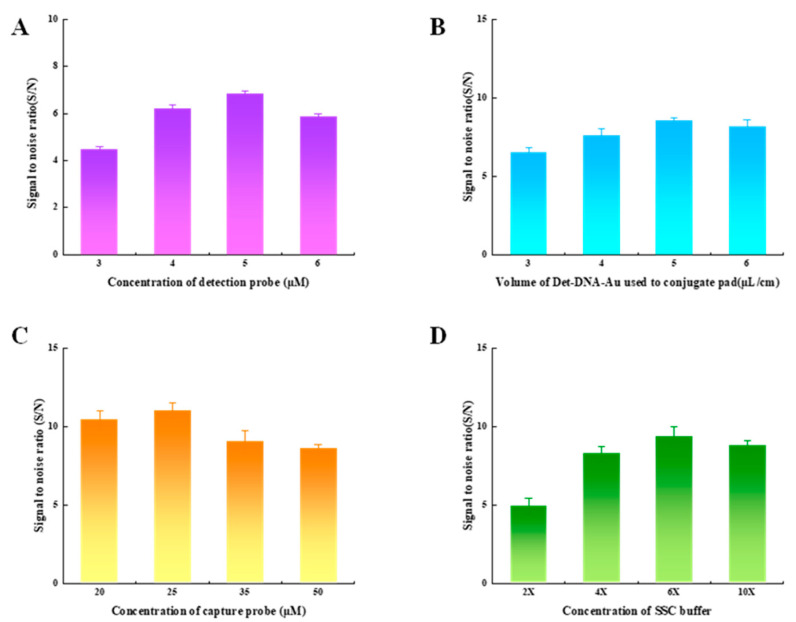
Optimization of experimental conditions: (**A**) concentration of Det-DNA, (**B**) volume of AuNP–Det-DNA conjugates, (**C**) concentration of Cap-DNA used to prepare the test zone, and (**D**) concentration of SSC. Error bars indicate the standard deviations of three independent measurements.

Four experimental parameters were optimized for the LFNAB preparation and DNA testing, including the optimal concentration of Det-DNA probe used to prepare AuNP–Det-DNA conjugates, the precise volume of Det-DNA–AuNPs dispensed on the conjugate pad, the appropriate concentration of Cap-DNA probe in the T zone, and the ideal concentration of SSC (saline sodium citrate buffer) in the running buffer. Four different concentrations of Det-DNA probes (3, 4, 5, and 6 μM) were used for the preparation of AuNP–Det-DNA conjugates. As shown in Figure 2A, the signal-to-noise (S/N) ratio increased with the increase in the Det-DNA concentration and reached the maximum at 5 μM, which was chosen for the preparation of AuNP–Det-DNA conjugates. The volume of Det-DNA–AuNPs conjugates used to prepare the LFNAB was optimized by dispensing different volumes of conjugate solution per strip. It was discovered that the highest S/N ratio was achieved when the volume sprayed on the conjugate pad was 5 μL (Figure 2B). Four different concentrations of Cap-DNA probe (20, 25, 35, and 50 μM) were used to prepare streptavidin–biotin–Cap-DNA complexes, which were distributed on the test zone of the LFNAB. As shown in Figure 2C, the highest S/N ratio was achieved at a Cap-DNA probe concentration of 25 μM. Running buffer has strong effect on the sensitivity of LFNABs, and SSC buffer has been proven to be one of the best buffers in DNA hybridization reactions. The effect of the SSC concentration on the S/N ratio of the LFNABs was studied. Four different concentrations of SSC (2×, 4×, 6×, 10×) were prepared. The highest S/N ratio was achieved when the SSC concentration was 6X (Figure 2D). In summary, 5 μM Det-DNA, 5 μL of AuNP–Det-DNA, 25 μM Cap-DNA, and 6× SSC were used as the best experimental conditions for the subsequent experiments.

### 2.3. Analytical Performances

**Figure 3:**
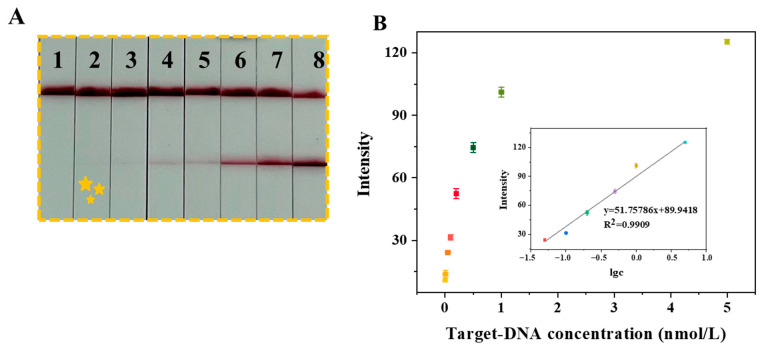
(**A**) Photo images of LFNABs after testing sample solutions containing different concentrations of target DNA (1: 0 nmol/L; 2: 0.005 nmol/L; 3: 0.01 nmol/L; 4: 0.05 nmol/L; 5: 0.1 nmol/L; 6: 0.5 nmol/L; 7: 1 nmol/L; 8: 5 nmol/L). (**B**) The resulting calibration curve. Assay time: 15 min; running buffer: 6× SSC. Yellow stars mark the visual detection limit. Error bars indicate the standard deviations of three independent measurements.

We tested the sample solutions with varying concentrations of target DNA under the best experimental conditions. Figure 3A shows photographs of the LFNABs after testing different target concentrations, ranging from 0 to 5 nM. The red band intensity in the test zones of the LFNABs increased as the target DNA concentration increased; no red band was observed in the test zone when the concentration of target DNA was 0 nM, indicating that there was no nonspecific adsorption. A weak band in the test zone was still visible even when the target DNA was lowered to 5 pM, which can be taken as the visual detection limit of the LFNAB. This detection limit is 100 times lower than that achieved with the salt-aging method (0.5 nM). The grayscale values of red bands in the T zones were analyzed using ImageJ software (v1.8.0.172) for quantitative analysis, and the resulting calibration plots of test band intensity versus DNA concentration were linear in the range of 0.005–5 nM (Figure 3B), which is suitable for quantitative work. The reproducibility of the LFNABs was evaluated by repeatedly testing samples at different concentration levels. The sample solutions with concentrations of 0.05 nmol/L and 0.5 nmol/L were selected for six determinations using the constructed LFNABs (the results of the determinations are shown in Figure S2), and the relative standard deviations were calculated to be 5.8% and 4.2%, respectively, indicating that the prepared LFNABs have good reproducibility.

**Figure 4:**
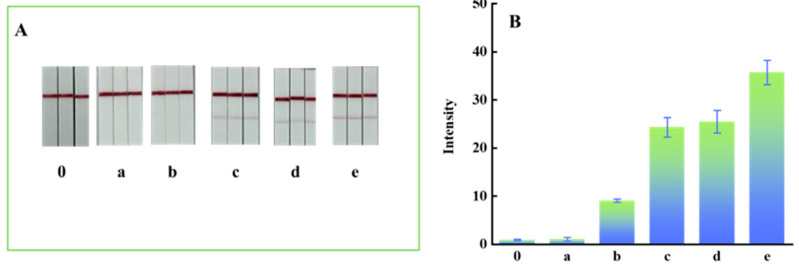
(**A**) Photo images of LFNABs after testing the sample solutions in the absence of target DNA (control, 0 pM) and in the presence of 100 pM non-complementary DNA (**a**), three-base mismatched DNA (**b**), two-base mismatched DNA (**c**), one-base mismatched DNA (**d**), and target DNA (**e**). (**B**) The corresponding intensity histogram. Assay time: 15 min. Error bars indicate the standard deviations of three independent measurements.

The specificity of LFNABs is a critical factor that determines the accuracy and reliability of the biosensors. We studied the specificity of the LFNABs by testing five DNA strands with different sequences at a concentration of 100 pM under the same conditions, including fully complementary target DNA, three-base mismatched DNA, two-base mismatched DNA, single-base mismatched DNA, and unrelated single-strand DNA. The test results are shown in Figure 4. No test band was observed when no target DNA existed, nor in the presence of 100 pM non-complementary DNA and three-base mismatched DNA; the test band intensity in the presence of 100 pM target DNA was significantly higher than that in the presence of two-base mismatched DNA and one-base mismatched DNA (Figure 4A). Figure 4B shows the corresponding intensity histogram of the test bands. The above results show that the optimized LFNAB under the butanol dehydration method has excellent specificity, enabling it to differentiate the fully complementary DNA and mismatched DNA.

### 2.4. Detecting miRNA-21 with the Optimized LFNAB

**Figure 5:**
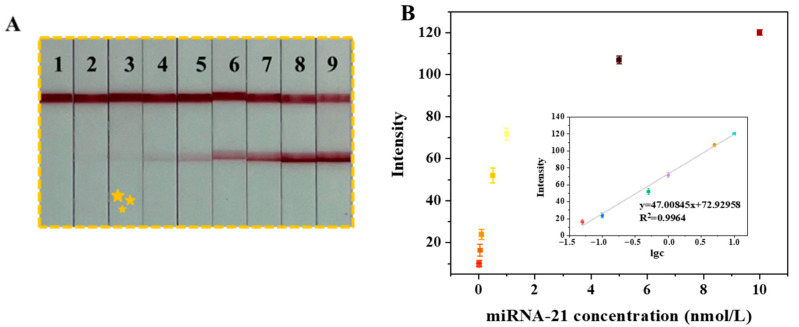
(**A**) Photo images of LFNABs after testing different concentrations of miRNA-21 (1: 0; 2: 0.01 nmol/L; 3: 0.02 nmol/L; 4: 0.05 nmol/L; 5: 0.1 nmol/L; 6: 0.5 nmol/L; 7: 1 nmol/L; 8: 5 nmol/L; 9: 10 nmol/L), and (**B**) the corresponding calibration curves for the miRNA-21 concentrations shown in (**A**). Inset: the linear range region. Error bars indicate the standard deviations of three independent measurements. Yellow stars mark the visual detection limit.

The optimized LFNAB was applied to detect microRNA-21 (miRNA-21) in pure buffer solution and spiked serum samples. Figure 5A shows photos of the LFNAB when testing different concentrations of miRNA-21. The signal strengths of the test bands increased with the increase in miRNA-21 concentration, and the visual detection limit was 20 pM. This detection limit is better than that of the reported AuNP-based lateral flow microRNA assay (Table 1). Figure 5B presents the connection between the test band intensity and miRNA-21 concentration. The inset in Figure 5B plots the variation of the intensity as a function of the concentration of miRNA-21, showing a good linear response for miRNA-21 concentrations in the range of 0.02–10 nmol/L (R^2^ = 0.9964).

The LFNAB was constructed to detect miRNA-21 in spiked serum samples. Solutions of serum with varying concentrations of miRNA-21 were prepared by introducing spiked miRNA-21 into 10-fold diluted serum solutions. The results are shown in Table 2. The recovery of the miRNA-21 assay was 94.78%~98.12%, with RSD values ranging from 1.16% to 2.24%. This result indicates that the LFNAB can be well applied to detect miRNA-21 in human serum.

## 3. Materials and Methods

### 3.1. Instrumentation and Reagents

The HM3030 XYZ three-dimensional film-spraying instrument, ZQ2002 microcomputer automatic chopping machine, and CTS350 CNC cutting machine were purchased from Shanghai Kinbio Tech. Co., Ltd. (Shanghai, China). The H3-16KR tabletop high-speed refrigerated centrifuge was purchased from Hunan Kecheng Instrument Equipment Co., Ltd. (Changsha, China), The MZC-2070M1 microwave oven was purchased from Haier Co., Ltd. (Qingdao, China). The HWS-24 Electric Thermostat Water Bath was purchased from Shanghai Huyan Industrial Co., Ltd. The refrigerator was purchased from Changhong Meiling Co., Ltd. (Hefei, China).

Trisodium phosphate, deoxyadenosine triphosphate (dATP), chloroauric acid (HAuCl_4_⋅4H_2_O), octylphenylpolyethylene glycol (Triton X-100), sodium dodecyl sulfate (SDS), phosphate-buffered saline (PBS), Tween 20, trisodium citrate, sucrose, bovine serum albumin (BSA), n-butanol, and poly(ethylene glycol) (PEG) 4000 were procured from Sinopharm Chemical Reagent Co., Ltd. (Shanghai, China); streptavidin (SA) was ordered from BBI Company. All oligonucleotide sequences are listed in the Supplementary Materials (Table S2). SX42 absorbent paper, CB06 glass-fiber paper, and an SMNF31-25 PVC bottom plate were bought from Shanghai Jinbiao Biological Co., Ltd. (Shanghai, China). CN140 nitrocellulose membrane was purchased from Sartorius Co., Ltd. (Goettingen, Germany).

### 3.2. Preparation of DNA–AuNP Conjugates

#### 3.2.1. Preparation of AuNPs

AuNPs with an average diameter of 15 ± 3.5 nm were synthesized by adopting the traditional method, with minor modifications [7]. Generally, 100 mL of HAuCl_4_ aqueous solution (0.01%) was added to a cleaned 500 mL conical bottle and placed on a magnetic stirrer for heating and stirring to boiling; then, 4.5 mL of trisodium citrate solution (1%) was introduced to the flask under stirring. The color of the solution gradually changed from colorless, to black, to dark red. After another 10 min of heating, the heating was stopped, and the solution was vigorously agitated for an additional 20 min. The prepared colloidal gold solution was then cooled down to the ambient room temperature. Subsequently, the solution was stored in a refrigerator, maintaining a temperature of precisely 4 °C. The as-prepared AuNPs were characterized by TEM, and the diameter of the AuNPs was around 15 ± 3.5 nm (Figure S3).

#### 3.2.2. Preparation of DNA–AuNP Conjugates

**Scheme 2:**
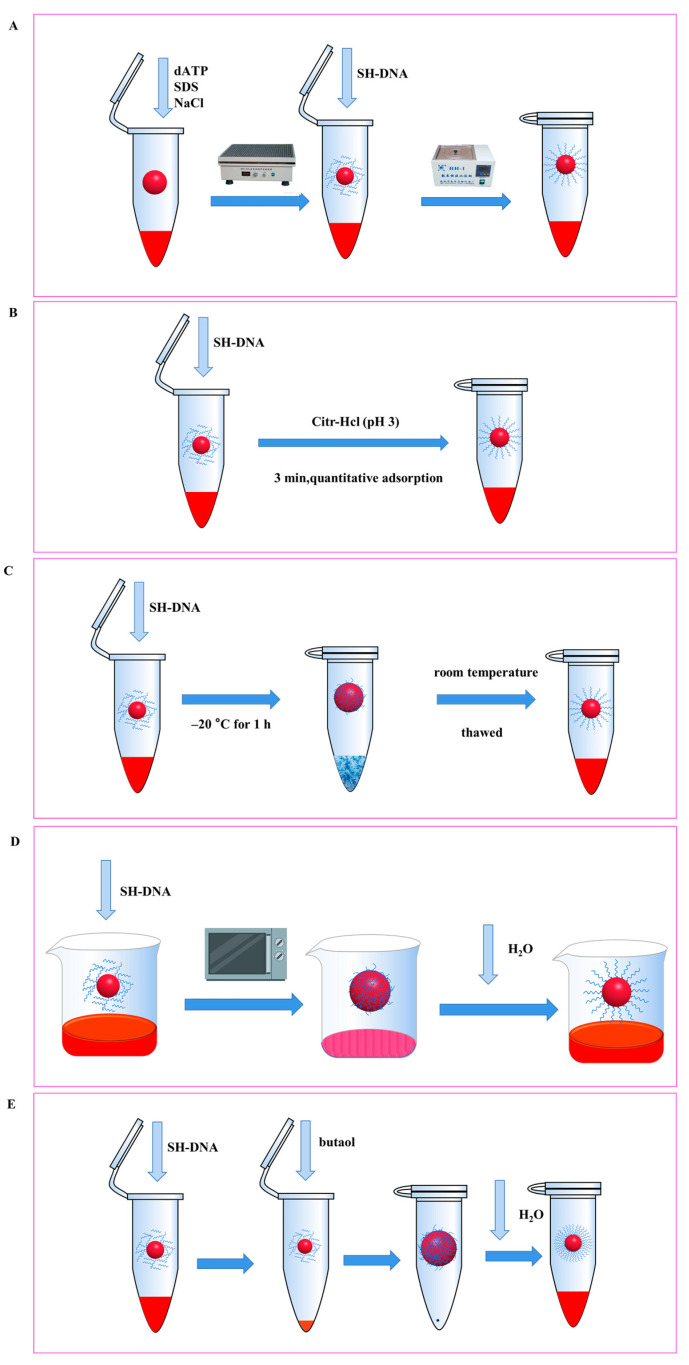
Preparation of AuNP–DNA conjugates: (**A**) salt-aging method; (**B**) low-pH method; (**C**) freeze–thaw method; (**D**) microwave dry heating method; (**E**) *n*-butanol dehydration method.

DNA–AuNP conjugates were prepared with five reported methods, with slight modifications (Scheme 2). The reaction conditions of the five methods for the preparation of DNA–AuNP conjugates are listed in Table 3.

##### Preparation of DNA–AuNP Conjugates Using the Salt-Aging Method (Scheme 2A) [32]

Five microliters (5 μL) of dATP at a concentration of 1 mmol/L was added to a 10-fold concentrated AuNP solution (100 μL) and incubated for 15 min. Afterwards, 7.5 μL of SDS (1%) was introduced twice, with a 5 min interval, followed by adding 25 μL of 2 mol/L NaCl. After the shielding reaction was completed, 5 μL of 100 μM SH-modified Det-DNA probe was pipetted into the AuNP solution. Then, the above solution was incubated at 60 °C for 3 h. The mixture was centrifuged for 10 min at 4 °C and 6000 rpm, and the red pellet was washed with PBS three times. Finally, the resulting DNA–AuNP conjugate was resuspended in 100 μL of eluent buffer (2.5% Tween 20, 10% sucrose, 5% BSA, and 20 mM Na_3_PO_4_) and stored at 4 °C prior to utilization.

##### Preparation of DNA–AuNP Conjugates Using the Low-pH Method (Scheme 2B) [33]

One hundred microliters (100 μL) of 10-fold concentrated AuNP solution was combined with 5 μL of Det-DNA probe at a concentration of 100 μM. After that, 2 µL of 0.5 M citrate buffer (pH 3.0) was incorporated into the solution to reduce its pH. To get the pH back to neutral, 3 μL of HEPES buffer (1 M, pH 7.6) was then added. Subsequently, the red pellet was washed with PBS 3 times and resuspended in 100 μL of eluent buffer.

##### Preparation of DNA–AuNP Conjugates Using the Freeze–Thaw Method (Scheme 2C) [34]

One hundred microliters (100 μL) of 10-fold concentrated AuNP solution was combined with 5 μL of Det-DNA probe at a concentration of 100 μM. The resulting mixture was subjected to freezing at a temperature of −20 °C for a duration of 1 h, after which it was thawed at RT. Following this, the particles were washed thrice with PBS. Finally, the resulting DNA–AuNP conjugate was resuspended in 100 μL of eluent buffer.

##### Preparation of DNA–AuNP Conjugates Using the Microwave-Assisted Dry Heating Method (Scheme 2D) [36]

A small beaker containing 100 μL of 10-fold concentrated AuNPs and 5 μL of 100 μM Det-DNA was placed in a microwave oven (input power 1150 W, output power 700 W) and heated in medium–high mode for 3 min, and the remaining solid on the bottom of the beaker was resuspended in 1 mL of PBS. The solution was centrifuged thrice at 12,400× *g* for 10 min at 15 °C to purify the AuNP–DNA conjugate, and then it was resuspended in 100 μL of eluent buffer. Finally, the conjugate was stored at 4 °C prior to utilization.

##### Preparation of DNA–AuNP Conjugates Using the Butanol Dehydration Method (Scheme 2E) [35]

The butanol dehydration method was conducted as previously reported, with minor modifications [35]. One hundred microliters (100 µL) of 10-fold concentrated AuNP solution was combined with 5 μL of Det-DNA probe at a concentration of 100 μM in the presence of 1 mM TCEP (Tris(2-carboxyethyl)phosphine). The mixture was supplemented with 600 µL of n-butanol, followed by rapid vortex-mixing (several seconds). The mixture was centrifuged at 2000× *g* for 10 s, and the upper phase was removed. The black pellet in the bottom of the centrifuge tube was resuspended in 100 μL of ddH_2_O. After centrifuging the solution at 8000× *g* and 4 °C for 10 min, the resulting pellet was washed three times with PBS to remove any remaining impurities. Finally, the resulting DNA–AuNP conjugate was resuspended in 100 μL of eluent buffer.

### 3.3. Assembly of the Lateral Flow Nucleic Acid Biosensor (LFNAB)

The LFNAB was composed of four parts from the till to the end—namely, the sample pad, DNA–AuNP conjugate pad, NC membrane, and absorbent pad (Scheme 1A). All parts were assembled on the PVC backing pad, and every two parts had a 2 mm overlap to ensure the progress of chromatography during detection. The AuNP–DNA conjugate solution was dispensed on the conjugate pad and desiccated at 37 °C for 2 h. Biotinylated Con-DNA or Cap-DNA probes (25 μM) were mixed with 1 mg/mL streptavidin (SA) at volume ratio of 1:3 for 2 h to form SA–biotin–DNA complexes. Then, the complex solution was dispensed on the NC membrane to form a control line and a test line with a 5 mm interval. Finally, the assembled strips were cut to a width of 2.98 mm and stored at 4 °C prior to utilization.

### 3.4. Lateral Flow Assay and Signal Analysis

A volume of 100 μL of running buffer spiked with different concentrations of target DNA or miRNA-21 was dropped onto the sample pad. After waiting for 15 min, a digital camera was used to take photo images of the LFNABs. The gray values of the test lines were analyzed using ImageJ.

### 3.5. Detection of microRNAs in Serum Samples

The LFNAB was utilized to detect microRNAs in serum samples to evaluate its potential for clinical application. Sample solutions with different concentrations of miR-21 were prepared by spiking miR-21 in 10-fold diluted serum in running buffer, and then tested as stated in Section 3.4.

## 4. Conclusions

In summary, the performances of lateral flow nucleic acid biosensors prepared with five AuNP–DNA conjugation methods were compared, and the butanol dehydration method offered the lowest detection limit. After systematic optimization, this LFNAB could detect target DNA at concentrations down to 5 pM without any signal amplification, and it also had excellent reproducibility and specificity. In addition, the LFNAB was applied to detect miRNA-21 with a detection limit of 20 pM, and it detected microRNA-21 in human serum with satisfactory results. However, the amount of butanol should be controlled appropriately in the process of dehydration. Too much butanol will lead to excessive dehydration and aggregation of conjugates, while too little butanol will lead to incomplete dehydration and affect the sensitivity of detection. Future work will aim to detect cancer-related microRNA biomarkers in patient samples and explore multiplex assays.

## Figures and Tables

**Table 1:** Comparison of the limits of detection of lateral flow microRNA biosensors.

Detection Target	Labels	LOD	Ref.
miRNA-21	AuNPs	115 pM	[37]
miRNA-21	AuNPs	70 pM	[38]
miRNA-21	RCA, AuNPs	40 pM	[39]
miRNA-215	AuNPs	60 pM	[40]
miRNA-31	AuNPs	33 pM	[41]
miRNA-21	AuNPs	20 pM	This work

**Table 2:** Results of the miRNA-21 recovery experiment (*n* = 3).

Sample Number	Spiked Concentration (nM)	Measured Concentration (nM)	Recovery (%)	RSD (%)
1	0.05	0.0481	96.2	2.24
2	0.5	0.4739	94.78	2.06
3	5	4.906	98.12	1.16

**Table 3:** Reaction conditions of the five methods for the preparation of DNA–AuNP conjugates.

Method	Concentrations of DNA (μM)	Volume of DNA (μL)	Concentrations of AuNPs (nM)	Volume of AuNPs (μL)	pH	Reaction Temperature	Buffer	Linkers
salt-aging	100	5	28	100	-	60 °C	-	dATP, thoil
low-pH	100	5	28	100	3	Room temperature	Citrate, HEPES	thoil
freeze-thaw	100	5	28	100	-	−20 °C	-	thoil
microwave-assisted heating dry	100	5	28	100	-	80–90 °C	-	thoil
butanol dehydration	100	5	28	100	-	Room temperature	-	thoil

## Data Availability

The data presented in this study are available in the article and in the Supplementary Materials.

## Supplementary Materials

The following supporting information can be downloaded at: https://www.mdpi.com/article/10.3390/molecules28114480/s1, Table S1: Performance of five methods for the preparation of DNA–AuNP complexes. Table S2: Sequences of nucleic acid used in this work. Figure S1: (A) UV–visible spectrum of AuNPs and AuNP–Det-DNA conjugates; (B) agarose gel electrophoresis images of AuNPs (left lane) and AuNP–Det-DNA conjugates (right lane). The concentration of agarose was 1.5%. Applied voltage: 100 V; electrophoresis time: 30 min. Figure S2: Photo images of LFNABs after testing 0.05 nmol/L target DNA (A) and 0.5 nmol/L target DNA (B); (C) the corresponding standard deviation histogram. Figure S3: (A) TEM image of AuNPs; (B) particle size distribution of AuNPs.

## References

[1] Zheng C., Wang K., Zheng W., Cheng Y., Li T., Cao B., Jin Q., Cui D. (2021). Rapid developments in lateral flow immunoassay for nucleic acid detection. Analyst.

[2] Lie P., Liu J., Fang Z., Dun B., Zeng L. (2012). A lateral flow biosensor for detection of nucleic acids with high sensitivity and selectivity. Chem. Commun..

[3] Hu J., Wang L., Li F., Han Y.L., Lin M., Lu T.J., Xu F. (2013). Oligonucleotide-linked gold nanoparticle aggregates for enhanced sensitivity in lateral flow assays. Lab Chip..

[4] Ngom B., Guo Y., Wang X., Bi D.R. (2010). Development and application of lateral flow test strip technology for detection of infectious agents and chemical contaminants: A review. Anal. Bioanal. Chem..

[5] Kumar P., Sarkar N., Singh A., Kaushik M. (2022). Nanopaper Biosensors at Point of Care. Bioconjug. Chem..

[6] Wang J., Zhu L.G., Li T.S., Li X.Y., Huang K.L., Xu W.T. (2022). Multiple functionalities of functional nucleic acids for developing high-performance lateral flow assays. TrAC Trends Anal. Chem..

[7] Mao X., Ma Y.Q., Zhang A.G., Zhang L.R., Zeng L.G., Liu G.D. (2009). Disposable nucleic acid biosensors based on gold nanoparticle probes and lateral flow strip. Anal. Chem..

[8] Xu Y., Liu Y.H., Wu Y., Xia X.H., Liao Y.Q., Li Q.G. (2014). Fluorescent probe-based lateral flow assay for multiplex nucleic acid detection. Anal. Chem..

[9] Mao X., Wang W., Du T.E. (2013). Dry-reagent nucleic acid biosensor based on blue dye doped latex beads and lateral flow strip. Talanta.

[10] Qiu W.W., Xu H., Takalkar S., Gurung A.S., Liu B., Zheng Y.F., Guo Z.B., Baloda M., Baryeh K., Liu G.D. (2015). Carbon nanotube-based lateral flow biosensor for sensitive and rapid detection of DNA sequence. Biosens. Bioelectron..

[11] Yu Q.C., Zhang J., Qiu W.W., Li K., Qian L.S., Zhang X.J., Liu G.D. (2021). Gold nanorods-based lateral flow biosensors for sensitive detection of nucleic acids. Microchim. Acta.

[12] Zhang J., Tang L.M., Yu Q.C., Qiu W.W., Li K., Cheng L.L., Zhang T.T., Qian L.S., Zhang X.J., Liu G.D. (2021). Gold-platinum nanoflowers as colored and catalytic labels for ultrasensitive lateral flow MicroRNA-21 assay. Sens. Actuators B Chem..

[13] Takalkar S., Baryeh K., Liu G.D. (2017). Fluorescent Carbon Nanoparticle-Based Lateral Flow Biosensor for Ultrasensitive Detection of DNA. Biosens. Bioelectron..

[14] Sapountzi E.A., Tragoulias S.S., Kalogianni D.P., Ioannou P.C., Christopoulos T.K. (2015). Lateral flow devices for nucleic acid analysis exploiting quantum dots as reporters. Anal. Chim. Acta.

[15] Lee K.X., Shameli K., Yew Y.P., Teow S.Y., Jahangirian H., Rafiee-Moghaddam R., Webster T.J. (2020). Recent developments in the facile bio-synthesis of gold nanoparticles (AuNPs) and their biomedical applications. Int. J. Nanomed..

[16] Yeh Y.C., Creran B., Rotello V.M. (2012). Gold nanoparticles: Preparation, properties, and applications in bionanotechnology. Nanoscale.

[17] Kohout C., Santi C., Polito L. (2018). Anisotropic gold nanoparticles in biomedical applications. Int. J. Mol. Sci..

[18] Saha K., Agasti S.S., Kim C., Li X., Rotello V.M. (2012). Gold nanoparticles in chemical and biological sensing. Chem. Rev..

[19] Gupta A., Moyano D.F., Parnsubsakul A., Papadopoulos A., Wang L.S., Landis R.F., Das R., Rotello V.M. (2016). Ultrastable and Biofunctionalizable Gold Nanoparticles. ACS Appl. Mater. Interfaces.

[20] Cutler J.I., Auyeung E., Mirkin C.A. (2012). Spherical nucleic acids. J. Am. Chem. Soc..

[21] Barnaby S.N., Perelman G.A., Kohlstedt K.L., Chinen A.B., Schatz G.C., Mirkin C.A. (2016). Design considerations for RNA spherical nucleic acids (SNAs). Bioconjug. Chem..

[22] Wang Z., Ma L. (2009). Gold nanoparticle probes. Coord. Chem. Rev..

[23] Kapadia C.H., Melamed J.R., Day E.S. (2018). Spherical nucleic acid nanoparticles: Therapeutic potential. BioDrugs.

[24] Kumar D., Saini N., Jain N., Sareen R., Pandit V. (2013). Gold nanoparticles: An era in bionanotechnology. Expert Opin. Drug Deliv..

[25] Sharifi M., Attar F., Saboury A.A., Akhtari K., Hooshmand N., Hasan A., El-Sayed M.A., Falahati M. (2019). Plasmonic gold nanoparticles: Optical manipulation, imaging, drug delivery and therapy. J. Control. Release.

[26] Elahi N., Kamali M., Baghersad M.H. (2018). Recent biomedical applications of gold nanoparticles: A review. Talanta.

[27] Goddard Z.R., Marín M.J., Russell D.A., Searcey M. (2020). Active targeting of gold nanoparticles as cancer therapeutics. Chem. Soc. Rev..

[28] Giljohann D.A., Seferos D.S., Prigodich A.E., Patel P.C., Mirkin C.A. (2009). Gene regulation with polyvalent siRNA−nanoparticle conjugates. J. Am. Chem. Soc..

[29] Huschka R., Zuloaga J., Knight M.W., Brown L.V., Nordlander P., Halas N.J. (2011). Light-induced release of DNA from gold nanoparticles: Nanoshells and nanorods. J. Am. Chem. Soc..

[30] Mirkin C.A., Letsinger R.L., Mucic R.C., Storhoff J.J. (1996). A DNA-based method for rationally assembling nanoparticles into macroscopic materials. Nature.

[31] Hurst S.J., Lytton-Jean A.K.R., Mirkin C.A. (2006). Maximizing DNA loading on a range of gold nanoparticle sizes. Anal. Chem..

[32] He Y., Zhang S., Zhang X., Baloda M., Gurung A.S., Xu H., Zhang X., Liu G. (2011). Ultrasensitive nucleic acid biosensor based on enzyme–gold nanoparticle dual label and lateral flow strip biosensor. Biosens. Bioelectron..

[33] Zhang X., Servos M.R., Liu J. (2012). Instantaneous and quantitative functionalization of gold nanoparticles with thiolated DNA using a pH-assisted and surfactant-free route. J. Am. Chem. Soc..

[34] Liu B., Liu J. (2017). Freezing directed construction of bio/nano interfaces: Reagentless conjugation, denser spherical nucleic acids, and better nanoflares. J. Am. Chem. Soc..

[35] Hao Y., Li Y., Song L., Deng Z. (2021). Flash synthesis of spherical nucleic acids with record DNA density. J. Am. Chem. Soc..

[36] Huang M., Xiong E., Wang Y., Hu M., Yue H., Tian T., Zhu D., Liu H., Zhou X. (2022). Fast microwave heating-based one-step synthesis of DNA and RNA modified gold nanoparticles. Nat. Commun..

[37] Kor K., Turner A.P.F., Zarei K., Atabati M., Beni V., Mak W.C. (2016). Structurally responsive oligonucleotide-based single-probe lateral-flow test for detection of miRNA-21 mimics. Anal. Bioanal. Chem..

[38] Chen T., Xu Y., Wei S., Li A., Huang L., Liu J.Q. (2018). A signal amplification system constructed by bi-enzymes and bi-nanospheres for sensitive detection of norepinephrine and miRNA. Biosens. Bioelectron..

[39] Yao M., Lv X., Deng Y., Rasheed M. (2019). Specific and simultaneous detection of micro RNA 21 and let-7a by rolling circle amplification combined with lateral flow strip. Anal. Chim. Acta.

[40] Gao X., Xu H., Baloda M., Gurung A.S., Xu L.P., Wang T., Zhang X., Liu G. (2014). Visual detection of microRNA with lateral flow nucleic acid biosensor. Biosens. Bioelectron..

[41] Sayers J., Payne R.J., Winssinger N. (2018). Peptide nucleic acid-templated selenocystine-selenoester ligation enables rapid miRNA detection. Chem. Sci..

