# Supplementary material for: Comparison of DNA–Gold Nanoparticle Conjugation Methods: Application in Lateral Flow Nucleic Acid Biosensors
Source: Molecules. 2023 Jun 1;28(11):4480. doi: 10.3390/molecules28114480 (PMC10254391; doi:10.3390/molecules28114480)
Supplement: Supplementary file 1 [file molecules-28-04480-s001.zip › molecules-2407495-supplementary.pdf]

Support Information

Comparison of DNA-Gold Nanoparticle Conjugation Methods: Application in Lateral Flow Nucleic Acid Biosensors

Qiaoling Ding [1, 2, 3] Wanwei Qiu [1, 4, *] Chunxue Sun [1, 2, 3] Hongxin Ren [3] Guodong Liu [1, 3, 5, *]

[1] College of Food Science, Anhui Science and Technology University, Fengyang, Chuzhou, Anhui, China

[2] Yangtze Delta Drug Advanced Research Institute, No.100, Dongtinghu Road, Linjiang Town, Haimen District, Nantong City, Jianshu, China

[3] Enfin Biotech (Jiangsu) Co. Ltd, No.100, Dongtinghu Road, Linjiang Town, Haimen District, Nantong City, Jianshu , China

[4] Institute of Biomedical and Health Science, School of Life and Health Science, Anhui Science and Technology University, Fengyang, 233100, Anhui, China

[5] School of Chemistry and Chemical Engineering, Linyi University, Shuangling Road, Linyi, 276000 Shandong, China

Table. S1 Performance of five methods for the preparation of DNA-AuNP conjugates

Method	Range	LOD(nM)	Sensitivity (nM)
salt-aging	0.1-10 nM	0.1	0.08
low-pH	0.1-10 nM	0.075	0.06
freeze-thaw	0.1-10nM	0.075	0.065
microwave-assisted heating dry	0.05-10 nM	0.05	0.04
butanol dehydration	0.005-5 nM	0.005	0.004

Table. S2 Sequences of nucleic acid used in this work

Nucleic acid	DNA/RNA Sequences (from 5 ' to 3 ')
Target DNA	TAGCTTATCAGACTGATGTTGA
Target miR-21	UAGCUUAUCAGACUGAUGUUGA
Detection DNA probe (Det-DNA)	SH-CCCCCTAGACACCGTGTTCAACATCAGT
Capture DNA probe (Cap-DNA)	CTGATAAGCTACCCCC-Biotin
Control DNAprobe (Con-DNA)	Biotin-ACACGGTGTCTAGGGGG
None complementary DNA	ATCGAATAGTCTGACTACAACT
Single-base mismatch	TAGCTTATCTGACTGATGTTGA
Two-base mismatch	TAGCTAATCAGACTGAAGTTGA
Three-base mismatch	TAGCTAATCAGTCTGAAGTTGA

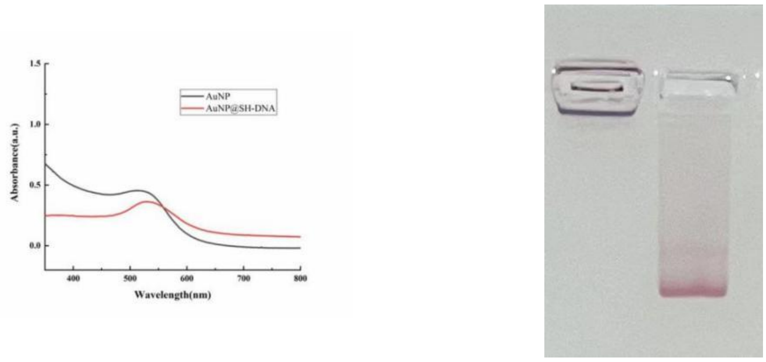

(A) (B)

Fig. S1 (A) UV-visible spectrum of AuNPs and AuNPs-Det-DNA conjugates; (B) Agarose gel electrophoresis image of AuNPs (left lane) and AuNPs-Det-DNA conjugates (right lane).The concentration of agarose was 1.5%. Applied voltage: 100 V; Electrophoresis time: 30 min.

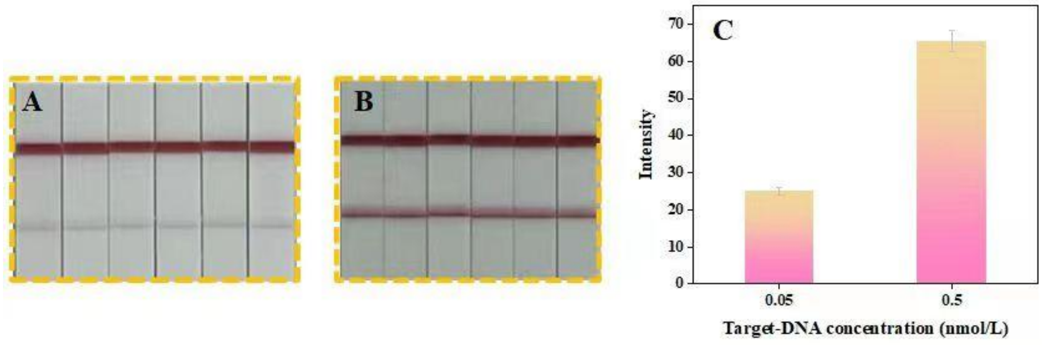

Fig. S2 Photo images of LFNABs after testing 0.05 nmol/L target DNA (A) and 0.5 nmol/L target DNA (B). (C) the corresponding standard deviation histogram.

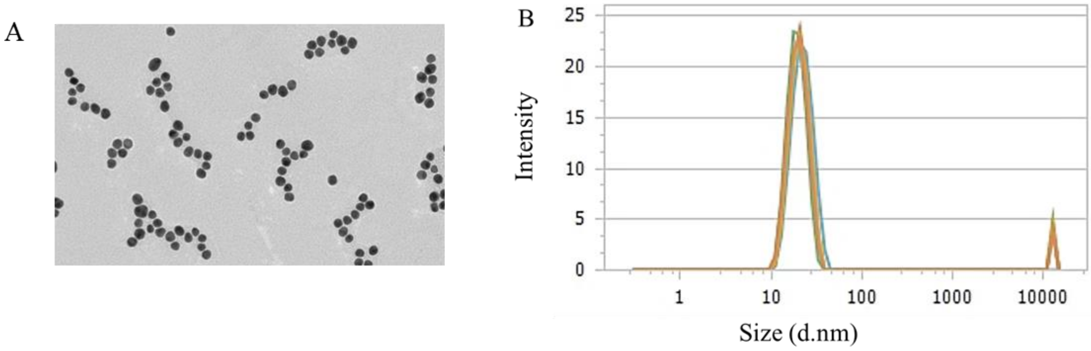

Fig. S3 (A) TEM image of AuNPs; (B) Particle size distribution of AuNPs
